# Carotid Sinus Syndrome in a Patient with Head and Neck Cancer: A Case Report

**DOI:** 10.7759/cureus.7042

**Published:** 2020-02-19

**Authors:** Manuel Toscano, Sérgio Cristina, Ana Rafaela Alves

**Affiliations:** 1 Internal Medicine, Hospital de Cascais, Cascais, PRT

**Keywords:** syncope, carotid sinus, head and neck neoplasms, radiotherapy

## Abstract

Syncope is a common complaint among patients presenting to the emergency department. Its differential diagnosis is broad and its management varies significantly depending on the underlying etiology. This is especially complex in patients with head and neck cancer since it may appear as an initial manifestation of the disease, as a side effect of surgery or radiotherapy, or as an indicator of local recurrence. Carotid sinus syndrome is a rare disease, whose pathophysiology is not yet fully understood. Here, we present the rare case of a 79-year-old male patient with a left cervical lymph node metastasis from an occult primary malignancy, who was admitted due to recurrent syncope. Paroxysms of extreme bradycardia were detected and a definitive dual chamber pacemaker was placed; however, the patient remained symptomatic. Cervical CT-scan revealed incarceration and compression of the left carotid sinus. The patient underwent radiotherapy, with favourable response, and remains asymptomatic to date.

## Introduction

Transient loss of conscience is a frequent cause of visit to healthcare services [1]. The differential diagnosis is extensive and includes heart and lung disease, metabolic disorders and epilepsy.

The carotid sinus integrates, along with the aortic arch bodies, the baroreceptor system, responsible for homeostasis and maintenance of blood pressure. Carotid sinus reflex receptors are found in the tunica adventitia and are responsible for generating impulses in response to arterial wall stretching, which are transmitted by the Hering nerve via the glossopharyngeal nerve to the solitary tract nucleus. Efferent fibres transmit adrenergic sympathetic innervation to the heart, resistance and capacitance vessels and vagus nerve, leading to increased parasympathetic flow, which results in bradycardia and transient arterial hypotension. Compromise of these neurally mediated mechanisms is a frequent and underdiagnosed cause of syncope [2].

In patients with head and neck cancer, syncope may appear as an initial manifestation of the disease, as a side effect of surgery or radiotherapy, or as an indicator of local recurrence [3]. Three syndromes, with distinct pathophysiology, are described in literature: carotid sinus syndrome (CSS), due to compression or invasion of the carotid sinus, which triggers an overactive reflex and strong efferent vagal response that may result in sinus bradycardia, sinus arrest or atrioventricular conduction disturbances; glossopharyngeal-asystole neuralgia syndrome, due to spontaneous afferent impulses of the glossopharyngeal nerve; and para-pharyngeal space syndrome, due to tumoral compression or invasion of the para-pharyngeal space and stimulation of glossopharyngeal afferent fibres. In addition, baroreceptor reflex failure syndrome is also described, usually secondary to surgery or radiotherapy.

There is general consensus in the definition of CSS, which reflects symptoms directly resulting from carotid sinus hypersensitivity (CSH). Although there is no universally accepted definition of CSH, the most commonly accepted one is presented by the 2017 American College of Cardiology/American Heart Association/Heart Rhythm Society (ACC/AHA/HRS) and the 2018 European Society of Cardiology (ESC) syncope guidelines, which consider an abnormal value to be heart rate (HR) pauses >3 seconds and a drop in systolic blood pressure (BP) >50 mmHg [1,4]. These values define the two main subtypes of CSS: cardioinhibitory (70-75% of cases) and vasodepressor (5-10%), respectively. A mixed subtype, combining both of these findings, may be present in 20-25% of patients.

CSS presents in older patients, with a mean age of 75 years, and has a strong male dominance (>2:1). It accounts for 1% of syncope cases [5]. Among these, head and neck tumours are rare (<1/250) and most cases occur in the presence of extensive cervical lymph node involvement [6,7]. Treatment depends on the frequency and severity of symptoms. Dual chamber pacing is most effective in patients with cardioinhibitory CSS and a negative tilt test. Therapy for the vasodepressor component of CSS, where tilt testing is likely positive, includes anticholinergics, fludrocortisone, midodrine and other vasopressors, but is often unsatisfactory [8]. Definitive treatment consists of surgical removal of the tumour. Other surgical methods include carotid sinus denervation by sectioning of the Hering nerve, arterial adventitial stripping or intracranial sectioning of the glossopharyngeal nerve and the first two roots of the vagus nerve, although there is insufficient data to prove the efficacy of these techniques [9].

## Case presentation

**Figure 1:**
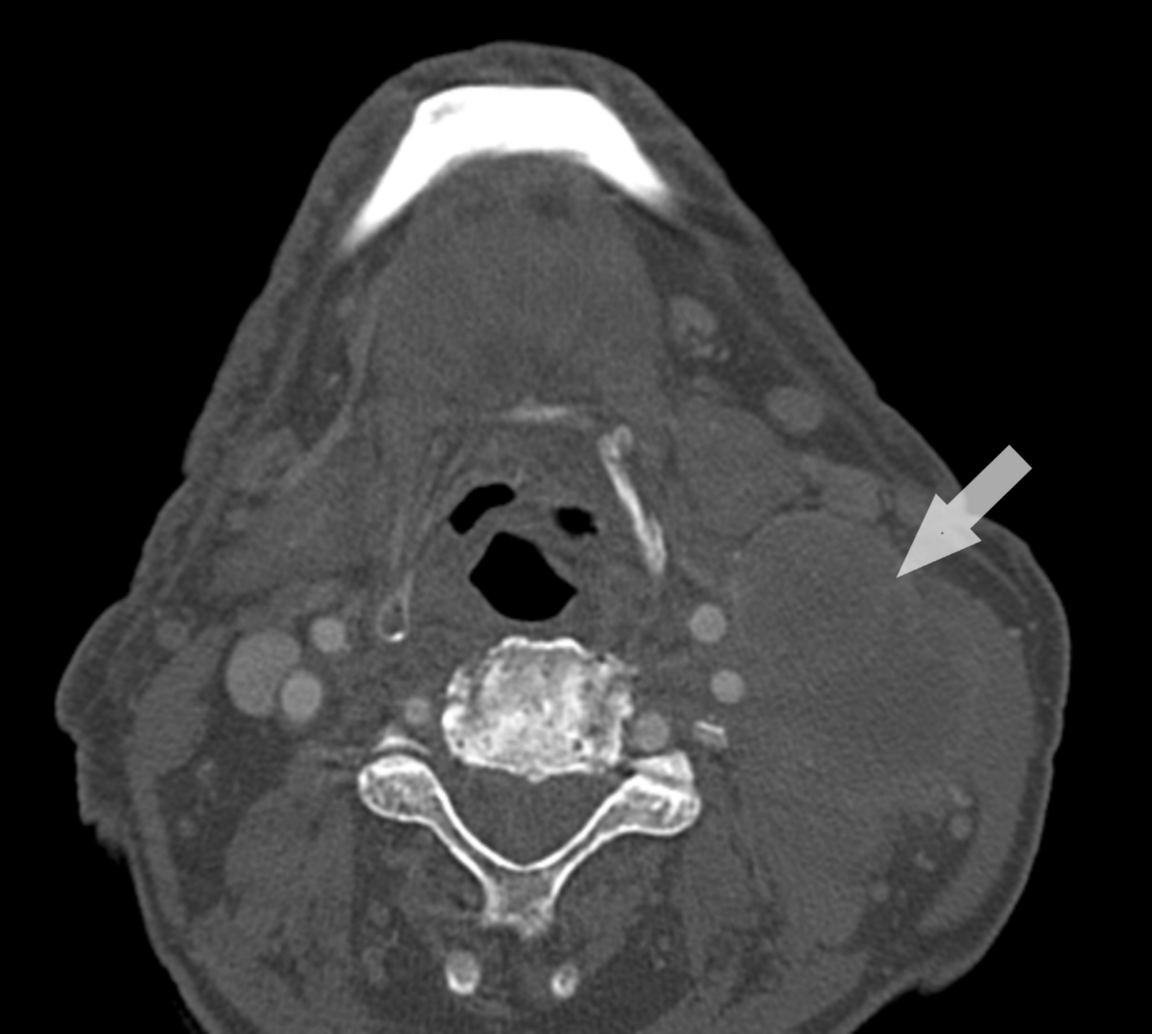
Cervical CT-scan at the time of diagnosis (axial view)

A 79-year-old male patient, with smoking habits and arterial hypertension, presented with mild weight loss and faintness for the past six months. A large left cervical mass was apparent and computerized tomography (CT) scan of the neck had revealed a large, solid, heterogenous lesion (47 x 35 mm), with several adjacent adenopathies (Figure 1).

CT-scan of the thorax, abdomen and pelvis showed an infracentimetric mass (5 mm) in the posterior segment of the left superior lobe, with no signs of metastasis. Intraoperatively, during incisional biopsy, infiltration of adjacent structures was observed. Immunohistochemistry was compatible with lymph node metastasis of a poorly differentiated CK7+/CK20- carcinoma, with focal marking for thyroid transcription factor 1 (TTF-1), suggestive of primary lung neoplasm. The disease was staged as cT0N2M0. Although the patient was referred to an Oncology consultation, follow-up was lost and no treatment was initiated.

Three months later, the patient presented once again due to recurrent syncope in the last month. He reported sudden episodes of blurry vision and dizziness, with no complaints of chest pain, dyspnea or palpitations, followed by loss of muscle tone and conscience, usually lasting less than 15 seconds, with full recovery in a few minutes. No neurologic deficits, orthostatic hypotension or other specific triggers were apparent. Junctional bradycardia (HR: 40-45 bpm) was detected, with no elevation of myocardial necrosis markers or electrolyte imbalance. Transthoracic echocardiography showed no abnormal findings and head-CT revealed only mild carotid atherosclerosis. Tilt testing was not performed. A diagnosis of sick sinus syndrome was assumed and a definitive DDDR pacemaker was placed.

**Figure 2:**
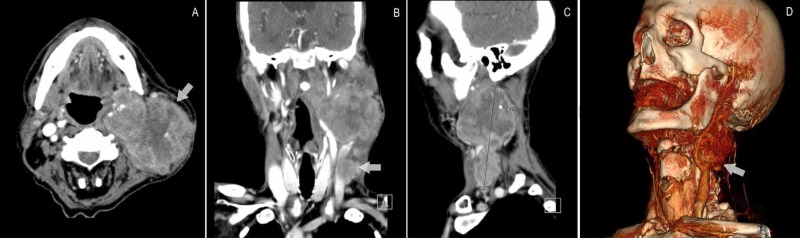
Cervical CT-scan on readmission, revealing incarceration of the left carotid sinus (A) Axial view; (B) coronal view; (C) sagittal view; (D) 3D reconstruction.

One week after discharge, the patient presented once again due to syncope. Physical examination and blood work remained unchanged. Pacemaker dysfunction was excluded. Soft tissue CT-scan of the neck was repeated, revealing a significant increase in tumour size (120 x 72 x 64 mm) and extensive infiltration of the sternocleidomastoid muscle and incarceration of the carotid bifurcation and internal and external carotid arteries, a finding not present in the previous study, which dated four months (Figure 2).

**Figure 3:**
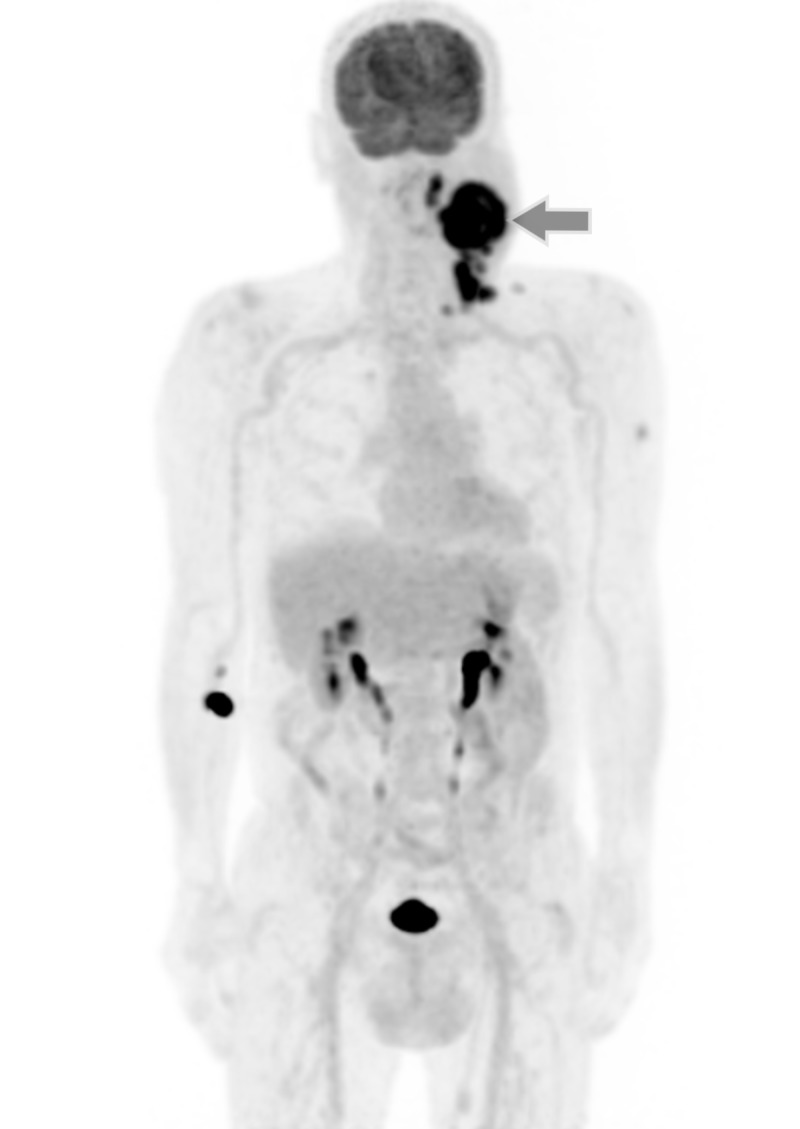
Positron emission tomography scan

Positron emission tomography (PET) scan showed viable neoplastic tissue in the large left latero-cervical mass, as well as in several other ipsilateral latero-cervical adenopathies in groups III, IV and V, with no other hypermetabolic activity, namely in the lung, and the tumour was re-staged as cT0N3M0 (Figure 3).

**Figure 4:**
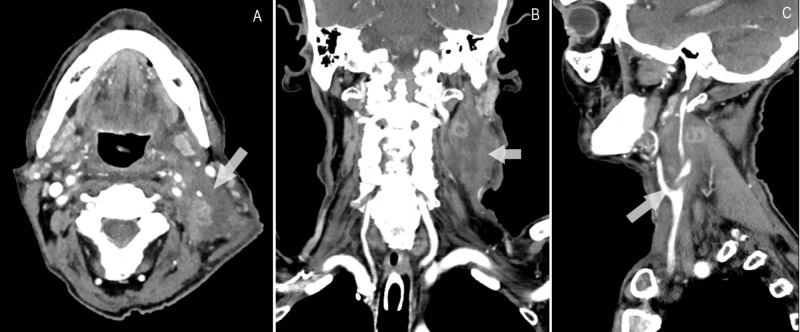
Cervical CT-scan after radiotherapy, showing significant decrease in mass size (A) Axial view; (B) coronal view; (C) sagittal view.

The patient was initiated on simultaneous integrated boost intensity modulated radiotherapy (SIB-IMRT), completing 33 sessions, with considerable decrease in tumour size (6 x 3 cm) (Figure 4).

No further episodes of lipothymia or syncope were noted and the patient remains asymptomatic to date, after one-year follow-up.

## Discussion

We present a case of carotid sinus syndrome due to compression and invasion of the carotid sinus by a lymph node metastasis of an occult carcinoma, which resulted in recurrent faintness and syncope. As described in most cases in literature, no precipitating factors were identified in this patient (i.e., hyperextension of the neck, constrictive clothing around the neck, coughing, sneezing or shaving).

Interestingly, although stimulation of both carotid sinuses causes a decrease in heart rate, in a study by Sigler with 345 healthy individuals, a difference in left and right carotid sinus stimulation was reported, the first being more sensitive to stimulation and more often associated with high-grade atrioventricular block (Mobitz II and third degree), while the latter usually results in sinus bradycardia [10]. Asystole was also twice as frequent with right stimulation when compared with left stimulation. In our case, as a result of compression of the left carotid sinus, only bradycardia was recorded and atrioventricular block was not observed. Carotid sinus response to stimulation is higher in men (4:1), as is the degree of heart rate decrease, especially in patients with atherosclerotic heart disease and hypertension, as in this case.

No specific therapy is recommended for patients with documented CSH without associated symptoms. Response to dual chamber pacing depends on the pathophysiology and predominant causative mechanism, with greater impact in select patients with episodes of asystole or severe bradycardia. As such, permanent pacemaker placement is indicated in patients with predominant or pure cardioinhibitory component (ACA/AHA/HRS class I indication; ESC class Ib indication) [1,4]. Despite this, studies show that 15% of patients have no clinical benefit and about 50% maintain mild or recurrent symptoms after pacemaker placement [11]. Furthermore, mixed subtype was associated with a two- to three-fold increase in the risk of symptom recurrence and several recent studies have shown no benefit in reducing syncope events and falls in elderly patients, contrary to what some previous small studies had indicated, such as SAFE PACE [12-14]. Persistence or predominance of the vasodepressor component has been implicated in these cases, which was also the most likely pathophysiologic mechanism in the patient described [15]. Spontaneous resolution has also been reported in rare cases.

It is important to note that, although severe bradycardia and hypotension may be present, the risk of mortality and cardiovascular events by CSH is not affected [16].

Definitive treatment consists in surgical removal of the tumour responsible for carotid sinus compression, when possible. Seeing as full surgical resection was not possible, and given the N3 staging, isolated radiotherapy was the safest option. Cisplatin radiosensitization was not performed because studies have shown no benefit in patients over 70 years [17,18].

## Conclusions

Unexplained syncope should alert the clinician to the possibility of carotid sinus compression, especially in patients with head and neck cancer. The treatment of recurrent reflex syncope in these patients is especially complex. Therapeutic options should be individualized according to the frequency and severity of symptoms and it is important to consider the risks associated with procedures for prevention of infrequent events.

## References

[1] Shen WK, Sheldon RS, Benditt DG (2017). 2017 ACC/AHA/HRS Guideline for the evaluation and management of patients with syncope: a report of the American College of Cardiology/American Heart Association Task Force on Clinical Practice Guidelines and the Heart Rhythm Society. Circulation.

[2] Crone RI, Massey FC (1948). Carotid sinus syndrome. Northwest Med.

[3] Newton HB, Malkin MG (2010). Neurological Complications of Systemic Cancer and Antineoplastic Therapy.

[4] Brignole M, Moya A, de Lange FJ (2018). 2018 ESC Guidelines for the diagnosis and management of syncope. Eur Heart J.

[5] Thomas JE (1969). Hyperactive carotid sinus reflex and carotid sinus syncope. Mayo Clin Proc.

[6] MacDonald DR, Strong E, Nielsen S, Posner JB (1983). Syncope from head and neck cancer. J Neurooncol.

[7] Papay FA, Roberts JK, Levine HL, Wegryn TL, Gordon T (1989). Evaluation of syncope from head and neck cancer. Laryngoscope.

[8] Sutton R (2014). Carotid sinus syndrome: progress in understanding and management. Glob Cardiol Sci Pract.

[9] Gardner RS, Magovern GJ, Park SB, Cushing WJ, Liebler GA, Hughes R (1975). Carotid sinus syndrome: new surgical considerations. Vasc Surg.

[10] Sigler LH (1934). Electrocardiographic observations on the carotid sinus reflex. Am Heart J.

[11] Healey J, Connolly SJ, Morillo CA (2004). The management of patients with carotid sinus syndrome: is pacing the answer?. Clin Auton Res.

[12] Parry SW, Steen N, Bexton RS, Tynan M, Kenny RA (2009). Pacing in elderly recurrent fallers with carotid sinus hypersensitivity: a randomised, double-blind, placebo controlled crossover trial. Heart.

[13] Ryan DJ, Nick S, Colette SM, Roseanne K (2010). Carotid sinus syndrome, should we pace? A multicentre, randomised control trial (Safepace 2). Heart.

[14] Kenny RA, Richardson DA, Steen N, Bexton RS, Shaw FE, Bond J (2001). Carotid sinus syndrome: a modifiable risk factor for nonaccidental falls in older adults (SAFE PACE). J Am Coll Cardiol.

[15] Gaggioli G, Brignole M, Menozzi C (1995). A positive response to head-up tilt testing predicts syncopal recurrence in carotid sinus syndrome patients with permanent pacemakers. Am J Cardiol.

[16] Hampton JL, Brayne C, Bradley M, Kenny RA (2011). Mortality in carotid sinus hypersensitivity: a cohort study. BMJ Open.

[17] Blanchard P, Landais C, Petit C (2016). Meta-analysis of chemotherapy in head and neck cancer (MACH-NC): an update on 100 randomized trials and 19,248 patients, on behalf of MACH-NC group. Ann Oncol.

[18] Lacas B, Bourhis J, Overgaard J (2017). Role of radiotherapy fractionation in head and neck cancers (MARCH): an updated meta-analysis. Lancet Oncol.

